# Non-Animal Models in Experimental Subarachnoid Hemorrhage Research: Potentials and the Dilemma of the Translation from Bench to Bedside

**DOI:** 10.1007/s12975-021-00950-0

**Published:** 2021-10-29

**Authors:** Cihat Karadag, Jay Gopalakrishnan, Christiane von Saß, Jan F. Cornelius, Daniel Hänggi, Jasper Hans van Lieshout, Marcel A. Kamp

**Affiliations:** 1 Department of Neurosurgery, Medical Faculty, Heinrich-Heine-University, Moorenstr. 5, 40225 Duesseldorf, Germany; 2 Institute of Human Genetics, Medical Faculty, Heinrich-Heine-University, Duesseldorf, Germany; 3 Department of Neurosurgery, Jena University Hospital, Friedrich-Schiller-University Jena, Am Klinikum 1, 07747 Jena, Germany

Dear Editor,

Worldwide, strokes are considered the second most prevalent cause of death. Around 7% are caused by subarachnoid hemorrhage (SAH), which in turn is associated with startlingly high morbidity and case fatality rate (CFR) of about 50% [1–3]. Translational approaches in the last decades have aimed to identify and establish new therapeutic approaches. Despite over 750 animal research studies having been published—some of which yielded promising results—translation from bench to bedside has largely failed so far [4, 5]. Potential reasons have previously been discussed extensively and include differences in brain and vessel anatomy, (patho-) physiology, genetics, and pharmaco-dynamics depending on the species and the drugs used [6–8]. Consensus definitions of important end points, such as delayed cerebral ischemia (DCI), are not generally used [9, 10]. Experimental SAH and SAH following aneurysm rupture in human beings often cannot be compared directly due to different pathophysiology and outcome parameters, e.g., vastly differing CFR [11]. Consequently, the use of animal models has been criticized for both scientific and ethical reasons.

Looking for a solution for this predicament, the suitability of (potential) non-animal experimental alternatives to study the complex pathophysiology after SAH has to be evaluated. However, so far, only a few preclinical in vitro models have been described for experimental SAH:

1. The most common preclinical SAH non-animal models are cell culture models. By growing individual cell populations, specific (cellular) pathomechanisms can be closely monitored, such as neuroinflammation, neuronal injury following SAH, and toxic side effects of blood and its components. However, when interpreting the results, the simplification of complex in vivo conditions has to be taken into account [12, 13].
2. A basic in vitro model of experimental SAH using blood, CSF, heme oxygenase-1, and rat arachnoid membranes was used to study the oxidation of unconjugated bilirubin by the cytochrome oxidase [14].
3. A computational analysis using a benchtop model consisting of a cranial vault attached to an idealized anatomical replica of the spinal canal allows the estimation of SAH clearance from cerebrospinal fluid (CSF) [15].

These—more or less advanced—cell cultures and basic in vitro models are promising tools that can lead to a more detailed understanding of the complex intracellular processes following SAH, e.g., to study changes in intracellular molecular pathways in response to SAH, increased pressure, or hypoxia. However, the pathophysiology of SAH is an orchestra of anatomical, mechanical, physiological, and molecular mechanisms, including impairments of cerebral perfusion, neuronal signaling and cellular energy balance, neuroinflammatory responses, and disruption of CSF circulation [16]. Simple cell culture and basic in vitro models clearly cannot depict these mechanisms—hence, more refined models are needed. An example of this represents the in vitro SAH—model of the perfused retina. The retinae used as a part of the central nervous system are usually a waste product generated in abattoirs since they cannot be further processed into food. Therefore, such retinae may serve as good to study neurovascular coupling and impairment of neuronal signaling by subarachnoid blood [17, 18]. Moreover, this model allows studying the impact of single cellular molecules or pathways for the pathophysiology of SAH [19].

Next to these established in vitro SAH models, some recent developments might be of great interest and potential for further SAH in vitro studies:

1. The brain-on-a-chip model allows the cultivation of different human cells in microfluidic chips. Using modifiable microchannels and microdomains inside the chip, a multicellular microenvironment that is able to imitate the neurophysiological conditions in the human brain with great accuracy can be created [20–22]. In the past few years, the brain-on-a-chip method allowed to depict neurophysiological processes and mechanisms of the human brain as well as pathological conditions [23–28]. Therefore, brain-on-a-chip models might be powerful tools for drug screening and disease modeling applications. So far, the brain-on-a-chip technique has not been used to model SAH, but it might be a promising approach in SAH research.
2. Embryonic stem cells are pluripotent in nature and capable of unlimited differentiation of any cell type. The possibility to create induced pluripotent stem cells (iPSCs) using artificial reprogramming of human somatic cells poses a modern possibility of modeling various organs in a preclinical environment. One such example is brain organoids which are complex and self-organizing three-dimensional (3D) systems that can be generated via differentiating iPSCs towards neuroectoderm lineages. These brain organoids are “brain-like neuroepithelial tissues” that constitute various cell types ranging from multipotent stem cells to layer-specific neurons whose cytoarchitecture is similar to its in vivo counterparts [29–32]. They show brain functionality and neurogenesis comparable to human neurophysiology [33, 34]. Generation of brain organoids with a vascular system is not possible since vasculature and endothelial cells originate from the mesoderm. Nevertheless, inducing a vascular system spontaneously within the brain organoids is essential for neurophysiological and neuroanatomic completion of organoid structures. Additionally, vascularization in brain organoids could enhance oxygen and nutrition supply, allowing culturing brain organoids for the long term. Recent efforts of co-culturing of endothelial cells and neurospheres resulted in the formation of vascularized brain organoids [35]. Further advancement was as induction of endothelial cells within brain organoids via an ectopic expression of ETS variant 2 (ETV2). This approach surprisingly generated functional vasculatures in brain organoids [36].

Taken together, brain organoids have the potential to fill the gap between in vitro cell cultures and in vivo animal models [37–40]. Indeed, Wang et al. [41] were able to use the self-organizing neural characteristic of brain organoids in a preclinical setting as part of the treatment of mice after SAH: comparing SAH-mice with and without organoid models, apoptosis was observed in less neurons when organoid models were implanted [41]. Furthermore, neurotransmitter-related neurons were formed due to synaptic connections between brain organoids and the host brain.

So far, neither a brain-on-a-chip nor a brain organoid model has been established to study SAH and further studies have to clarify as to how far they are suitable to portray the human SAH pathophysiology and to develop new therapeutic approaches. In contrast to animal models, brain organoids have the advantage to offer personalized brain organoids when generated from iPSCs derived from individual patients. These organoids, as part of individualized medicine, may completely portray the individual patient with his genetic composition [42]. Therefore, these brain organoids will at least complement in vivo animal studies. Taken together with recently developed non-mammal animal models (e.g., zebrafish), this might substitute “classical” animal models in preclinical stroke research as discussed above [43, 44].

In conclusion, there exists a dilemma in translational SAH research: new therapeutic approaches are urgently needed but translation from bench to bedside with animal models has largely failed. Cell culture and basic in vitro models likely enable an analysis of some intracellular processes but are unable to give a complete picture. More advanced models are promising but have not yet been established except for the perfused retina model.

Consequently, future research has to focus on the refinement of sophisticated in vitro SAH models, such as a brain-on-a-chip or a brain organoid model. As in vitro approaches will unlikely replace animal experiments completely, differences in the pathophysiology of SAH between different species must be analyzed and standardization in experimental SAH has to be aimed for.
